# PATZ1 is a new prognostic marker of glioblastoma associated with the stem-like phenotype and enriched in the proneural subtype

**DOI:** 10.18632/oncotarget.19546

**Published:** 2017-07-25

**Authors:** Elia Guadagno, Michela Vitiello, Paola Francesca, Gaetano Calì, Federica Caponnetto, Daniela Cesselli, Simona Camorani, Giorgio Borrelli, Marialuisa Califano, Paolo Cappabianca, Claudio Arra, Elvira Crescenzi, Laura Cerchia, Maria Laura Del Basso De Caro, Monica Fedele

**Affiliations:** ^1^ Department of Advanced Biomedical Sciences, Pathology Section, University of Naples Federico II, 80131 Naples, Italy; ^2^ Institute of Experimental Endocrinology and Oncology (IEOS) “Gaetano Salvatore”, National Council of Research, 80131 Naples, Italy; ^3^ Department of Medical and Biological Sciences, University of Udine, 33100 Udine, Italy; ^4^ Division of Neurosurgery, Department of Neurosciences, Reproductive and Odontostomatological Sciences, University of Naples Federico II, 80131 Naples, Italy; ^5^ Department of Experimental Oncology, National Cancer Institute “Fondazione Giovanni Pascale”, IRCCS, 80131 Naples, Italy

**Keywords:** glioma, glioma stem cells, PATZ1, tumor heterogeneity, biomarker

## Abstract

Glioblastoma (GBM), the most malignant of the brain tumors, has been classified on the basis of molecular signature into four subtypes: classical, mesenchymal, proneural and neural, among which the mesenchymal and proneural subtypes have the shortest and longest survival, respectively. Here we show that the transcription factor *PATZ1* gene is upregulated in gliomas compared to normal brain and, among GBMs, is particularly enriched in the proneural subtype and co-localize with stemness markers. Accordingly, in GBM-derived glioma-initiating stem cells (GSCs) PATZ1 is overexpressed compared to differentiated tumor cells and its expression significantly correlates with the characteristic stem cell capacity to grow as neurospheres *in vitro*. Interestingly, survival analysis demonstrated that PATZ1 lower levels informed poor prognosis in GBM and, specifically, in the proneural subgroup, suggesting it may serve a role as diagnostic and prognostic biomarker for intra-subtype heterogeneity of proneural GBM. We also show that PATZ1 suppresses the expression of the mesenchyme-inducer CXCR4, and that PATZ1 and CXCR4 are inversely correlated in GSC and proneural GBM. Overall these findings support a central role of PATZ1 in regulating malignancy of GBM.

## INTRODUCTION

Glioblastoma (GBM) is the most frequent primary malignant brain tumor and is one of the most aggressive neoplasms among the human cancers [1]. It is characterized by dismal prognosis with median survival of 16–19 months despite multimodal treatment including surgical resection followed by combined radiochemotherapy [2]. In spite of enormous efforts towards understanding the molecular basis of the disease and the development of novel therapeutic strategies, only limited advances have been achieved and there is a pressing need to identify proteins and signalling pathways that can serve as new targets for an improved treatment of GBM. The identification of brain tumour stem-like cells [3], in both adult and pediatric malignant gliomas, has provided new insights into the biology of malignant glial tumours rendering these cells a potentially valuable therapeutic target. Indeed, a major drawback in the treatment of GBM is its resistance against standard therapeutic approaches, and current data attribute the resistance against both γ-irradiation and chemotherapeutic alkylating agent Temozolomide to the cancer stem cells [4]. Therefore, therapeutic targets in glioma-initiating stem cells (GSCc) are a focus of increasing interest to improve the GBM outcome.

Systematic gene expression and sequencing of GBM (WHO grade IV gliomas) have revealed a great heterogeneity of this disease with at least two major tumor subgroups (proneural and mesenchymal) and two other intermediate possible groups (classical/proliferative and neural) [5–7]. Among them, the proneural subtype is characterized by a stem-like signature and cells with a full stem phenotype [8], TP53 mutations and unresponsiveness to aggressive chemo- and radio- treatments [7].

All normal tissues host a small population of stem cells. These are “initiating cells’ with the unique ability to proliferate and differentiate into a variety of other cells depending on the tissue in which they are located in the body. In the brain, neogenesis of mature cells persists throughout adult life within discrete regions, primarily in the dentate gyrus of the hippocampus and in the subventricular zone of the forebrain lateral ventricles. The emerging hypothesis is that alterations in the cellular and genetic mechanisms that control adult neurogenesis might contribute to brain tumorigenesis. The main implication of continued adult neurogenesis is the presence of undifferentiated, mitotically active stem and progenitor cells within discrete regions of the mature brain. These populations might function as a source of cells for transformation, giving rise to GSCs [9]. Despite the stem cell population within a tumour represents a minor subpopulation (only 2% of cancer cells), it is responsible for tumor maintenance and progression, and it phenocopies the original tumour in rodent xenograft models. Depletion of the cancer stem cell population greatly impairs the potential of the bulk tumours to initiate xenograft tumour formation and leads to prolonged survival of tumour-bearing mice [10]. While the suitability of GSCs as therapeutic targets has been recently validated *in vivo* [11], the molecular signalling pathways orchestrating the biology of GSCs, as well as of the other cancer stem cells, remain to be elucidated.

It has been recently shown that the C2H2 zinc finger protein PATZ1 (POZ, AT-hook and Zinc-finger 1) is an important regulator of pluripotency in embryonic stem cells, directly involved in the regulation of pluripotency master genes, such as Pou5f1 and Nanog, which together with Sox2 are necessary for maintaining self renewal and pluripotency [12]. A PATZ1 role in neural stem cells has also been suggested by our previous studies showing it is highly expressed in actively proliferating neuroblasts of the periventricular and subventricular neocortical neuroepithelium, and Patz1-knockout mice have proliferative defects of the subventricular zone [13]. PATZ1 is a member of the POK (POZ and kruppel-like zinc finger) family, a unique group of transcription factors playing key roles in development and cancer through their involvement in a variety of cellular processes, including cell proliferation, senescence and apoptosis [14]. PATZ1 itself has been implicated in all these processes [13, 15–20]. In addition, several studies indicate a role of PATZ1 in carcinogenesis, sometimes working as a tumour suppressor and sometimes as an oncogene, depending on the cellular context [16–23]. In human brain tumour cells PATZ1 has been found to be included in the SOX2-interactome [24], and targeting of PATZ1 by siRNA in human GBM cell lines resistant to conventional chemotherapy, increases their sensitivity to apoptotic stimuli [17].

Here we characterized the expression of PATZ1 in human gliomas showing it is overexpressed compared to normal brain and is significantly enriched in the proneural GBM subtype, where it behaves as a valuable prognostic marker. We also show that PATZ1 protein in GBM co-localizes with stem cell markers, is increased in GBM-derived stem cells compared with tumor differentiated cells, and correlates with the capacity of these cells to grow as spheres. Finally, we show that in GBM and GBM-derived cells PATZ1 expression negatively correlates with the expression of the G-protein coupled receptor and mesenchyme inducer CXCR4, suggesting a role of PATZ1 in suppressing the mesenchymal subtype.

## RESULTS

### PATZ1 is expressed in human gliomas and is enriched in the proneural subtype

Using paraffin embedded sections generated from surgical specimens collected at the Department of Advanced Biomedical Sciences, Pathology Section, of the University of Naples Federico II, we analysed by immunohistochemistry PATZ1 expression in 45 grade IV GBM, 22 oligodendrogliomas (10 grade III and 12 grade II) and 26 perilesional normal brain parenchymas. Of 45 GBM patients included in the study, 30 were males and 15 females, aged between 26 and 81 years. The lesion was mainly localized in the temporal lobe in 22 cases, parietal in 11 cases, frontal in 9 cases, occipital in 2 cases and 1 in the corpus callosum (Supplementary Table 1). Of 22 oligodendroglioma patients 15 were males and 7 females, aging from 22 to 69 years. 14 cases were frontal, 4 temporal, 3 occipital and 1 was an intramedullary metastasis (Supplementary Table 2).

**Figure 1:**
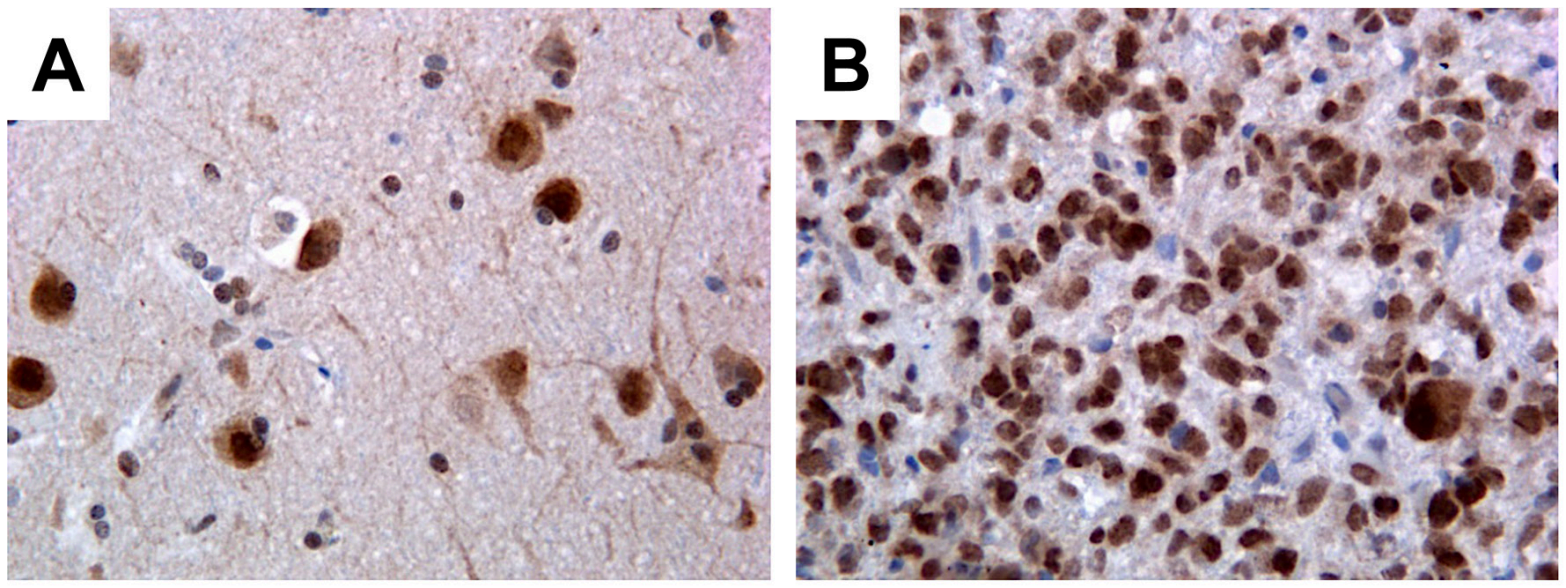
Immunohistochemical detection of PATZ1 in human GBM and perilesional normal cortex **(A)** Perilesional normal cortex: only neurons stain positively, mainly in the nucleus, while glial cells are negative. **(B)** Representative GBM: most of neoplastic cells are positive. Original magnification: 40x.

**Figure 2:**
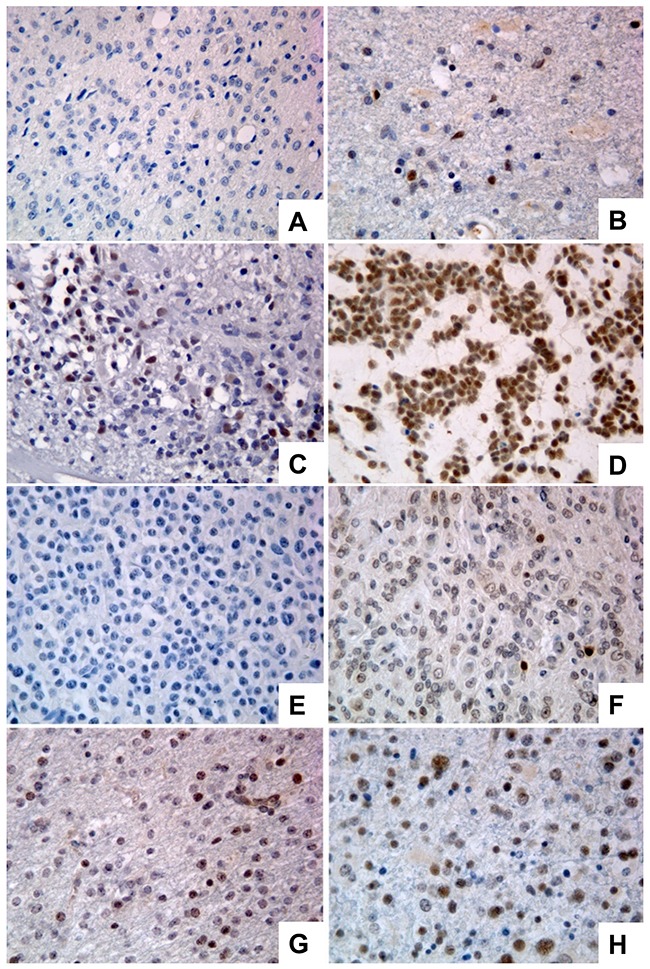
Immunoreactivity score in GBMs and oligodendrogliomas stained for PATZ1 **(A)** Representative GBM scored 0. **(B)** Representative GBM scored +. **(C)** Representative GBM scored ++. **(D)** Representative GBM scored +++. **(E)** Representative oligodendroglioma scored 0. **(F)** Representative oligodendroglioma scored +. **(G)** Representative oligodendroglioma scored ++. **(H)** Representative oligodendroglioma scored +++. Original magnification: 40x.

We have observed that PATZ1 protein is expressed in neurons (mainly in the nucleus) but not in glial cells of all perilesional normal brain tissues (Figure 1A), whereas it is expressed in a high percentage of GBM (69%) with a main nuclear, but sometimes cytoplasmic localization (Figure 1B, Supplementary Table 1) and oligodendrogliomas (54%) (Supplementary Table 2). Among positive samples, PATZ1 immunoreactivity was variable in both GBM and oligodendrogliomas, including samples scored + (< 10%), ++ (>10% <50%), +++ (≥ 50%). Of 31 GBM positive for PATZ1 staining, 17 displayed +, 9 ++ and 5 +++ score (Figure 2A–2D, Supplementary Table 1). Among the 12 oligodendrogliomas expressing PATZ1, 4 cases had +, 5 ++ and 3 +++ score (Figure 2E–2H, Supplementary Table 2). Among the assessed clinicopathological features of GBM (age, gender, site), a high significant correlation and a trend to be correlated with PATZ1 expression was present in the male gender (p = 0.0028) and younger patients (p = 0.0695), respectively. Moreover, a statistically significant correlation was observed between PATZ1 immunoreactivity and grading (p = 0.0286) in oligodendrogliomas (Table 1).

**Table 1:** Fisher's exact test: clinicopathological features and PATZ1 expression

Variables			PATZ1 expression	
GBM	n.	+/++/+++	-	p
**Age**	45			
<60	11	10 (91%)	1 (9%)	0.0695
≥60	34	21 (62%)	13 (38%)	
**Gender**	45			
Males	30	27 (90%)	3 (10%)	**0.0028**
Females	15	7 (47%)	8 (53%)	
**Site**	42			
Temporal	22	14 (64%)	8 (36%)	0.5586
Parietal	11	9 (82%)	2 (18%)	
Frontal	9	6 (67%)	3 (33%)	
**Oligodendrogliomas**				
**Age**	22			
<60	17	9 (53%)	8 (47%)	0.7805
≥60	5	3 (60%)	2 (40%)	
**Gender**	22			
Males	15	9 (60%)	6 (40%)	0.4520
Females	7	3 (43%)	4 (57%)	
**Site**	21			
Frontal	14	6 (43%)	8 (57%)	0.1974
Temporal	4	2 (50%)	2 (50%)	
Occipital	3	3 (100%)	0 (0%)	
**Grading**	22			
II	12	4 (33%)	8 (67%)	**0.0286**
III	10	8 (80%)	2 (20%)	

**Figure 3:**
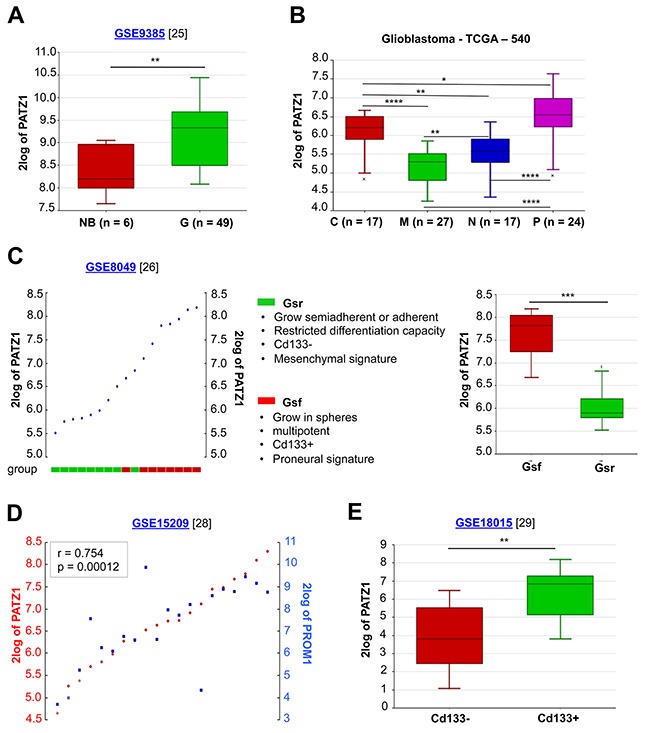
*In silico* meta-analysis of *PATZ1* expression in human GBM tissues and cell lines **(A)** Box-plot comparing PATZ1 expression in normal brain (NB) and gliomas of different grade (G). The data were analyzed by one-way analysis of variance (ANOVA) through the R2 web platform. **(B)** Box-plot showing PATZ1 expression in the different GBM subtypes and statistical analysis of the differences among them. C = classical, M = mesenchymal, N = neural, P = proneural. The data were analyzed by one-way analysis of variance (ANOVA) through the R2 web platform. **(C)** On the left, YY-plot in GSCs (ordered by PATZ1 expression). The cells are divided in two group as indicated in the figure. On the right, box-plot comparing PATZ1 expression in the two groups. **(D)** YY-plot correlating *PATZ1* and *PROM1* expression in stem cells derived from GBM (n = 7), oligoastrocytoma (n = 2), normal human foetus (n = 5) and primary biopsies of non-malignant brain tissue (n = 6). Significance of correlation (inbox) was analyzed by Pearson's χ^2^ test through the R2 web platform (http://r2.amc.nl). **(E)** Box-plot comparing PATZ1 expression in primary GBM sorted for Cd133 expression (negative/positive). The data were analyzed by one-way analysis of variance (ANOVA) through the R2 web platform. *, p < 0.05; **, p < 0.01; ***, p < 0.001; ****, p < 0.0001. Datasets for each cohort of samples are indicated in the figure with the relative reference in brackets. 2log of PATZ1 = logarithmic values of PATZ1 expression with base of 2.

By analysing public datasets through the R2: genomic Analysis and Visualization platform (http://r2.amc.nl), we confirmed in an independent sample cohort of 6 adult non diseased brains, 26 GBMs and 21 oligodendrogliomas [25] that *PATZ1* is significantly more expressed in gliomas (including GBMs and oligodendrogliomas) compared to non diseased brain samples (Figure 3A). Moreover, in a data set from the Tumor Cancer Genomic Atlas (TCGA), including 85 samples, sub-classified in classical (n = 17), mesenchymal (n = 27), neural (n = 17) and proneural (n = 24) GBM, we found that *PATZ1* expression specifically correlates with the molecular subtype, being highest in proneural and lowest in mesenchymal subtype, with significant differences between each subtype (Figure 3B). Consistently, by studying the gene signature correlated with the expression of *PATZ1* in the context of GBM, we found that PATZ1 expression positively associates with proneural genes, including *SOX2*, *OLIG2*, *DLL3*, *NOTCH1*, *PDGFRα* and many others, while negatively associates with the mesenchymal marker CD44 and many other genes characterizing the mesenchymal signature (Table 2 and Supplementary Table 3).

**Table 2:** Correlations between *PATZ1* and the proneural and mesenchymal signature in GBM^a^

Proneural signature			Mesenchymal signature		
Gene	r^b^	p	gene	r	p
*CHD7*	0.649	3.9e-62	*HEXB*	-0.597	3.8e-50
*CSNK1E*	0.633	3.1e-58	*SQRDL*	-0.583	2.7e-47
*MARCKSL1*	0.592	4.4e-49	*TMBIM1*	-0.537	4.1e-39
*SOX4*	0.580	7.1e-47	*IL15*	-0.535	7.1e-39
*NOTCH1*	0.574	1.0e-45	*RAB32*	-0.528	1.0e-37
*MAP2*	0.564	9.4e-44	*ANXA4*	-0.524	5.1e-37
*SOX11*	0.557	1.1e-42	*S100A4*	-0.523	5.7e-37
*DLL3*	0.549	3.8e-41	*FCGR2B*	-0.522	1.0e-36
*PODXL2*	0.545	1.3e-40	*RRAS*	-0.518	4.4e-36
*MMP16*	0.539	1.4e-39	*P4HA2*	-0.513	2.2e-35
*FXYD6*	0.535	6.9e-39	*VAMP5*	-0.505	3.8e-34
*TOP2B*	0.532	1.8e-38	*NPC2*	-0.499	2.7e-33
*PHF16*	0.532	2.2e-38	*CAST*	-0.497	6.3e-33
*SEZ6L*	0.526	2.1e-37	*PLS3*	-0.497	4.7e-33
*BCAN*	0.524	4.0e-37	*COPZ2*	-0.495	1.1e-32
*CRMP1*	0.519	3.1e-36	*PLAUR*	-0.495	9.4e-33
*NLGN3*	0.518	4.2e-36	*CLEC2B*	-0.490	5.4e-32
*WDR6*	0.511	3.9e-35	*LGALS3*	-0.477	3.9e-30
*PHLPP1*	0.500	1.8e-33	*LY96*	-0.476	5.6e-30
*MTSS1*	0.494	1.5e-32	*CLIC1*	-0.475	6.1e-30
*OLIG2*	0.494	1.6e-32	*PIGP*	-0.473	1.1e-29
*NRXN2*	0.488	1.2e-31	*ALOX5*	-0.466	1.0e-28
*PAFAH1B3*	0.488	1.2e-31	*TNFAIP8*	-0.465	1.3e-28
*DBN1*	0.484	4.1e-31	*CSTA*	-0.461	4.3e-28
*MMP15*	0.483	5.8e-31	*S100A11*	-0.461	5.0e-28
*FLRT1*	0.479	1.8e-30	*CTSB*	-0.453	4.9e-27
*DPF1*	0.479	1.9e-30	*CASP4*	-0.453	4.5e-27
*ASCL1*	0.479	1.7e-30	*ANXA2*	-0.447	2.9e-26
*CRB1*	0.476	5.3e-30	*RAB27A*	-0.442	1.1e-25
*DCX*	0.472	1.5e-29	*MGST2*	-0.440	1.9e-25
*SATB1*	0.472	1.6e-29	*SP100*	-0.439	2.7e-25
*ZNF510*	0.471	2.1e-29	*ARPC1B*	-0.437	4.6e-25
*RUFY3*	0.467	7.5e-29	*TLR2*	-0.437	4.4e-25
*GNG4*	0.467	7.8e-29	*ASL*	-0.433	1.6e-24
*MYT1*	0.461	5.1e-28	*KYNU*	-0.433	1.3e-24
*CASK*	0.451	9.9e-27	*PROCR*	-0.433	1.7e-24
*CDK5R1*	0.446	4.4e-26	*FHL2*	-0.433	1.4e-24
*VAX2*	0.442	1.1e-25	*LGALS1*	-0.432	2.1e-24
*GSK3B*	0.436	5.9e-25	*TIMP1*	-0.432	1.8e-24
*NCAM1*	0.430	3.7e-24	*PHF11*	-0.432	2.1e-24
*MAPT*	0.429	4.5e-24	*MAN1A1*	-0.425	1.3e-23
*CKB*	0.425	1.4e-23	*AMPD3*	-0.424	1.5e-23
*CBX1*	0.425	1.3e-23	*POLD4*	-0.418	7.2e-23
*KIF21B*	0.425	1.3e-23	*CYBRD1*	-0.418	7.1e-23
*MYO10*	0.424	1.6e-23	*CHI3L1*	-0.418	7.9e-23
*NOL4*	0.423	2.4e-23	*IFI30*	-0.416	1.2e-22
*MPPED2*	0.422	2.8e-23	*FCGR2A*	-0.414	2.1e-22
*BCOR*	0.420	5.4e-23	*ACSL1*	-0.413	3.0e-22
*CSPG5*	0.419	6.0e-23	*MAN2A1*	-0.413	2.7e-22
*CLASP2*	0.414	2.3e-22	*SYPL1*	-0.412	3.7e-22
*HMGB3*	0.406	1.5e-21	*IQGAP1*	-0.405	2.4e-21
*TMCC1*	0.403	3.3e-21	*RAC2*	-0.403	3.6e-21
*NKX2-2*	0.400	7.1e-21	*ICAM3*	-0.402	4.3e-21
*RALGPS1*	0.400	7.1e-21	*MS4A4A*	-0.401	6.6e-21
*SOX2*	0.399	9.7e-21	*CRYZ*	-0.401	6.2e-21
*GRIA2*	0.399	1.0e-20	*ANXA1*	-0.400	8.2e-21
*TTYH1*	0.397	1.4e-20	*PTGER4*	-0.396	2.1e-20
*ZNF184*	0.397	1.5e-20	*LTBP2*	-0.396	2.2e-20
*SCN3A*	0.396	1.9e-20	*SLC16A3*	-0.395	2.4e-20
*KLRC3*	0.394	3.2e-20	*NCF2*	-0.393	3.8e-20
*AMOTL2*	0.391	6.6e-20	*TGFBI*	-0.391	6.2e-20
*TAF5*	0.389	1.0e-19	*MAFB*	-0.390	7.6e-20
*DUSP26*	0.383	3.8e-19	*RHOG*	-0.388	1.4e-19
*RAP2A*	0.383	4.4e-19	*CTSC*	-0.387	1.5e-19
*CDC7*	0.380	8.1e-19	*SWAP70*	-0.386	2.2e-19
*SRGAP3*	0.377	1.7e-18	*IGFBP6*	-0.383	4.1e-19
*WASF1*	0.375	2.4e-18	*SERPINA1*	-0.382	5.3e-19
*ICK*	0.372	5.2e-18	*MFSD1*	-0.382	5.6e-19
*RAD21*	0.371	6.4e-18	*ACPP*	-0.381	6.1e-19
*GSTA4*	0.368	1.1e-17	*TNFAIP3*	-0.380	8.9e-19
*SEC61A2*	0.367	1.5e-17	*IL1R1*	-0.380	8.3e-19
*EPHB1*	0.364	2.8e-17	*SYNGR2*	-0.377	1.6e-18
*DPYSL4*	0.363	3.3e-17	*CASP8*	-0.376	1.9e-18
*NRXN1*	0.360	6.5e-17	*C5AR1*	-0.375	2.4e-18
*PDGRA*	0.352	3.6e-16	*CTSZ*	-0.375	2.6e-18
*ERBB3*	0.352	3.8e-16	*ARHGAP29*	-0.369	9.6e-18
*ABAT*	0.350	5.3e-16	*SHC1*	-0.366	1.8e-17
*GADD45G*	0.345	1.4e-15	*SERPINE1*	-0.366	1.7e-17
*CDC25A*	0.341	3.7e-15	*MSR1*	-0.366	1.9e-17
*ATP1A3*	0.338	5.8e-15	*FXYD5*	-0.365	2.2e-17
*YPEL1*	0.336	9.6e-15	*THBD*	-0.364	2.8e-17
*FBXO21*	0.335	1.1e-14	*LAIR1*	-0.363	3.6e-17
*ARHGEF9*	0.335	1.1e-14	*CD14*	-0.359	8.9e-17
*HDAC2*	0.331	2.2e-14	*GRN*	-0.358	1.1e-16
*ZNF248*	0.328	4.3e-14	*PTPRC*	-0.354	2.6e-16
*FHOD3*	0.324	8.5e-14	*LY75*	-0.353	3.1e-16
*HOXD3*	0.320	1.9e-13	*GNA15*	-0.350	5.6e-16
*TSAPN3*	0.318	2.8e-13	*VDR*	-0.349	7.1e-16
*TOPBP1*	0.318	3.0e-13	*CASP1*	-0.349	6.3e-16
*EPB41L5*	0.317	3.1e-13	*ITGB2*	-0.346	1.4e-15
*BEX1*	0.316	3.9e-13	*PTPN22*	-0.345	1.7e-15
*DGKI*	0.314	5.7e-13	*EMP3*	-0.345	1.7e-15
*STMN1*	0.314	6.1e-13	*TCIRG1*	-0.344	1.8e-15
*CA10*	0.308	1.7e-12	*SLAMF8*	-0.344	2.0e-15
*MCM10*	0.307	1.9e-12	*SIGLEC7*	-0.344	1.7e-15
*P2RX7*	0.305	3.0e-12	*ITGAM*	-0.338	5.7e-15
*PDE10A*	0.295	1.6e-11	*SCPEP1*	-0.336	8.8e-15
*FGF9*	0.293	2.2e-11	*TGFBR2*	-0.333	1.5e-14
*RAB33A*	0.293	2.1e-11	*STAT6*	-0.333	1.6e-14
*TMEM35*	0.290	3.6e-11	*LILRB2*	-0.332	2.1e-14
*GRID2*	0.290	3.6e-11	*SIGLEC9*	-0.330	3.0e-14
*PPM1D*	0.288	4.8e-11	*PLAU*	-0.324	8.6e-14
*PAK7*	0.286	6.6e-11	*ARSJ*	-0.324	9.5e-14
*NANOG*	0.284	1.0e-10	*SEC24D*	-0.323	1.1e-13
*PPM1E*	0.280	1.8e-10	*TRIM38*	-0.319	2.3e-13
*MAST1*	0.279	2.2e-10	*ELF4*	-0.318	2.7e-13

^a^ All the genes belong to either the proneural or mesenchymal signatures as described by Verhaak et al. [7].

^b^ Correlations were analyzed by Pearson's χ^2^ test through the R2 platform (http://r2.amc.nl). For more correlations between *PATZ1* and the mesenchymal signature see Supplementary Table 5.

Taken together protein and gene expression data, PATZ1 is overexpressed in gliomas compared to normal glia and appears as a specific biomarker of the proneural GBM subtype.

### PATZ1 is preferentially expressed in glioma stem cells

It has been recently reported that a proneural-like gene expression signature is characteristic of GSCs characterized by a full stem phenotype (Gsf lines), growth as floating spheres, CD133 expression and multipotency, while a mesenchymal-like gene expression signature is associated with GSCs characterized by a restricted stem phenotype (Gsr lines), adherent growth, no CD133 expression and limited differentiation [8, 26, 27]. Consistently, by analysing the expression profiles of the 9 GSCs and relative sub-clones isolated and characterized by Günter and colleagues (4 with Gsf phenotype and 5 with Gsr phenotype) we found that PATZ1 is significantly enriched in Gsf-GSCs compared with Gsr-GSCs (Figure 3C). The association of PATZ1 expression with CD133 expression was further confirmed by the analysis of two separate experimental datasets consisting of cell lines established from 7 GBMs, 2 oligoastrocytomas and 5 normal foetuses plus primary biopsies from 6 normal brains [28], and 8 fresh primary and non cultured GBMs [29], where *PATZ1* expression is highly correlated with that of *PROM1* (the gene encoding prominin-1/CD133) (Figure 3D), and is significantly enriched in the CD133+ compared to the CD133- subpopulation (Figure 3E). Consistently, in these cells lines *PATZ1* also correlates with markers of oligodendrocyte precursor cells, including *SOX8*, *OLIG1*, *OLIG2*, and *NKX2.2*, and of the proneural phenotype, such as *DLL3*, *OLIG2* and *SOX2* (Supplementary Table 4). All together these results indicate that PATZ1 is preferentially expressed in GSCs in comparison with matched non-GSCs, and is particularly enriched in proneural *versus* mesenchymal subtype.

**Figure 4:**
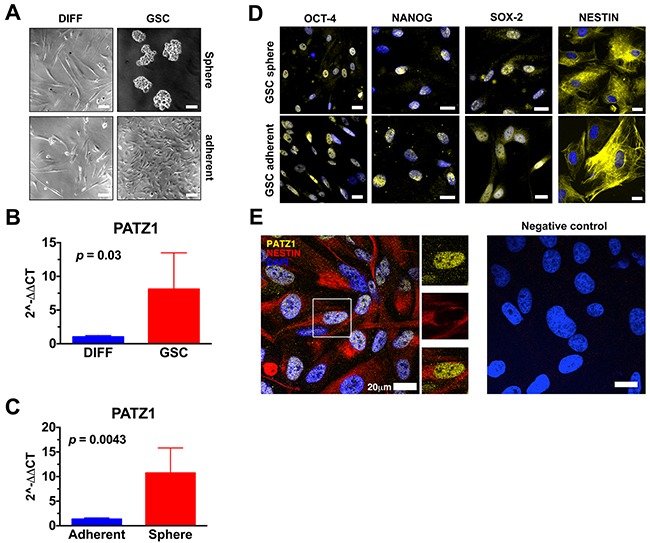
PATZ1 expression in GSC cultures **(A)** Representative phase contrast pictures of human glioma cells cultured in media selective (GSC, right panels) or not (DIFF, left panels) for the growth of glioma stem cells. The upper-right panel displays GSC growing as neurosphere, while the lower-right panel displays GSC growing in adhesion. Scale bar: 100μm. **(B)** Gene expression changes of *PATZ1* in human glioma cells grown in media selective (GSC) or not (DIFF) for the growth of GSC, as assessed by qRT-PCR. Data are presented as mean ± SD. **(C)** Gene expression changes of *PATZ1* in GSC growing in adhesion (adherent) or in suspension (sphere), as assessed by qRT-PCR. Data are presented as mean ± SD. **(D)** Representative immunofluorescence images depicting in yellow the expression of OCT4, NANOG, SOX-2, and NESTIN in GSC growing either in adhesion (GSC adherent, lower panels) or in suspensions (GSC sphere, upper panels). Nuclei are depicted by the blue fluorescence of DAPI. Scale bar: 20 μm. **(E)** Representative confocal immunofluorescence image (merge image) showing the expression of PATZ1 (yellow fluorescence) and Nestin (red fluorescence) in GSCs. Nuclei are depicted by the blue fluorescence of DAPI. Single immunodetection of either PATZ1 or Nestin in the inset area, and digital merge of the images, are shown in the middle. Negative control obtained by omitting the primary antibodies is shown on the right. Scale bar: 20 μm.

To validate the *in silico* results, we analyzed *PATZ1* expression in 6 GSCs (3 growing in adhesion and 3 growing as spheres in laminin-coated dishes) isolated from patients affected by GBM in comparison with matched non-stem tumor cells (Figure 4A). The results confirmed an enriched expression of *PATZ1* in stem *versus* non-stem matched samples (P < 0.05; Figure 4B). The differences do not reach the statistical significance if we consider each group (sphere forming and growing in adhesion GSCs) singularly, likely because of the low number of samples, but they have a trend to be higher in GSCs compared to matched non-stem tumor cells (data not shown). Moreover, *PATZ1* expression was also analyzed in 5 more GBM-derived GSCs, for a total of 11 GSCs, which were sub-divided in two groups on the basis of their growth capacity in laminin-coated dishes (sphere/adherent). As shown in Figure 4C, and consistently with data shown in Figure 3C, *PATZ1* expression was highly enriched in GSCs growing as spheres compared to GSCs growing adherent (P < 0.01). All isolated GSCs, independently from their modality to grow in laminin-coated dishes, expressed markers of stemness, as confirmed by immunofluorescence analysis (Figure 4D), at higher levels compared with matched DIFF cells (data not shown). Immunofluorescence analysis confirmed expression of PATZ1 in GSC growing either as spheres or in adhesion and their colocalization with the stemness marker Nestin (Figure 4E and data not shown). PATZ1 expression here appears mainly nuclear but a speckled cytosolic expression can be observed as previously described in other cancer cell types [20–22].

We also used the GBM cell lines U87MG to isolate GSC and analyze PATZ1 expression in comparison with matched bulk and differentiated cells (Supplementary Figure 1). PATZ1 protein expression resulted enriched in GSC *versus* both bulk and differentiated matched cells and correlated with expression of platelet-derived growth factor receptor β (PDGFRβ), a stem cell-specific marker in human GBM [30].

**Figure 5:**
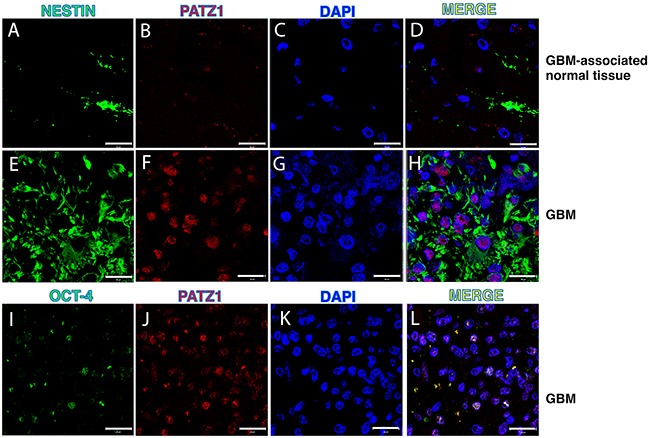
PATZ1 localization in the stem cell compartment of GBM Representative confocal microscopy images of GBM (two samples with high PATZ1 score (+++) and GBM-associated normal tissues stained with anti-NESTIN **(A, E)**, anti-OCT-4 **(I)** and anti-PATZ1 **(B, F, J)**. **(D, H, L)** Merge images showing co-localization of PATZ1 with either NESTIN or OCT-4. Scale bar: 20 μm. **(C, G, K)** Nuclei are depicted by the blue fluorescence of DAPI.

To further confirm *in vivo* the association of PATZ1 with the stem cell compartment of the GBM, we performed immunofluorescence labeling with antibodies anti-PATZ1 and GSC markers, including NESTIN and OCT-4, in 5 GBMs positive for PATZ1 expression. As shown in Figure 5, PATZ1 (red nuclear signal) was co-expressed with NESTIN (green cytoplasmic signal) in a sub-population of neoplastic cells. In these cells co-localization of OCT-4 (green nuclear signal) with PATZ1 was also evident.

### PATZ1 expression levels predict survival of GBM patients

**Figure 6:**
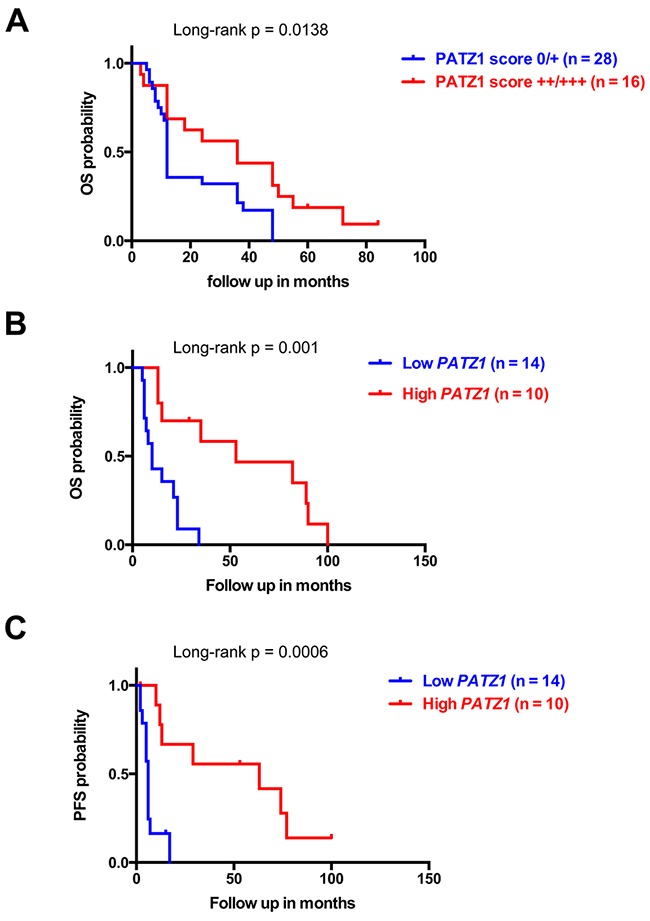
PATZ1 expression negatively correlates with worse survival in GBM patients **(A)** Overall (OS) Kaplan-Meier survival curves of GBM patients shown in Supplementary Table 1 stratified by protein levels of PATZ1, as indicated. **(B)** OS and **(C)** progression-free (PFS) Kaplan-Meier curves of the proneural subset of GBM patients from the TCGA data set shown in Figure 4B. Note that patients with lower PATZ1 expression have a worse survival rate than patients with higher PATZ1 as assessed by log-rank test.

The proneural GBM subtype is characterized by a trend toward longer survival, but is totally unresponsive to aggressive treatment with concurrent chemo- and radiotherapy [7]. Consistent with the enrichment of PATZ1 in the proneural subtype, we showed in our local cohort of gliomas, that patients with low PATZ1 score (0/+) had a worse overall survival (OS) than patients with high PATZ1 score (++/+++) (HR = 2.525, 95% CI = 1.208-5.280, p = 0.0138) (Figure 6A). The median OS for 16 patients with high PATZ1 was 36 months, in contrast to 12 months for 28 patients with low or absent PATZ1. Similarly, when we extended survival analyses to a larger cohort of GBM samples (n = 85), in which all subtypes were similarly represented (GBM – TCGA - 540), both OS and progression-free survival (PFS) were significantly correlated to PATZ1 expression, being worse in patients expressing low levels of PATZ1 (Supplementary Figure 2). In this same cohort of samples proneural GBMs have longer survival compared with mesenchymal subtype (Supplementary Figure 2, Panel C). Interestingly, also when we restricted the analysis to the proneural subset of GBM, Kaplan-Meier survival curves, followed by log-rank test, showed that PATZ1 expression stratifies these patients (n = 24) in two statistically different subgroups where low PATZ1 has worse OS (HR = 3.191, 95% CI = 2.355-14.31, p = 0.001) and PFS (HR = 3.595, 95% CI = 2.785-19.06, p = 0.0006) (Figure 6B–6C). The median OS for 10 patients with high *PATZ1* expression was 53 months, in contrast to 10 months for 14 patients with low *PATZ1*. Even more strikingly, the median PFS for patients with high *PATZ1* was 63 months *versus* 6 months for patients with low *PATZ1*. Moreover, the 5-year PFS was about 40% for proneural GBM patients with high *PATZ1* versus 0 with low *PATZ1*. In the same group of patients, using a multivariate Cox regression analysis, including known prognostic factors, such as age at diagnosis and methylation of the MGMT gene, low expression of PATZ1 resulted an independent prognostic factor for OS survival (HR= 0.2886, 95% CI = 0.0982-0.8482, p = 0.0246) and PFS (HR = 0.2458, 95% CI = 0.0802-0.7534, p = 0.0146).

These data suggest that PATZ1 expression has a crucial impact on outcome of GBM patients with a proneural classification, and could be used as an independent prognostic marker to predict survival and response to treatment.

### PATZ1 downregulates *CXCR4* expression in GBM

**Figure 7:**
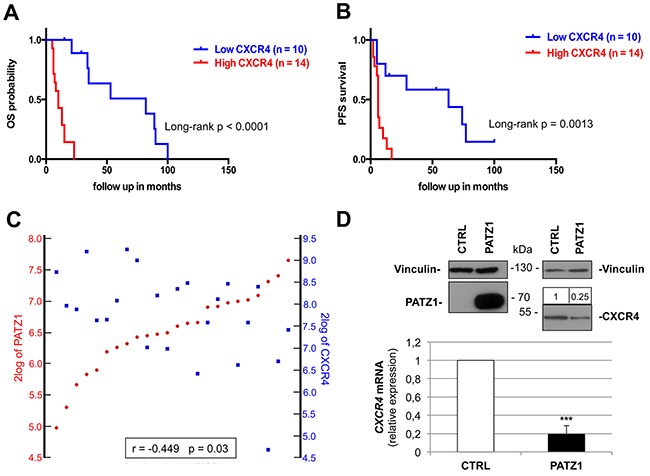
*CXCR4* expression correlates positively with worse survival and negatively with *PATZ1* in proneural GBM patients **(A)** Overall (OS) and **(B)** progression-free (PFS) Kaplan-Meier survival curves of the patients shown in Figure 7B-7C (proneural subset of the TCGA dataset of GBM). Note that patients with higher *CXCR4* expression have worse survival rates than patients with lower *CXCR4*, as assessed by log-rank test. **(C)** YY-plots correlating *PATZ1* and *CXCR4* expression in the proneural GBMs shown in A and B. Significance of correlation (inbox) was analyzed by Pearson's χ^2^ test through the R2 web platform (http://r2.amc.nl). **(D)** VS-GB cells were transiently transfected with 10 μg PATZ1 expressing plasmid, and endogenous CXCR4 mRNA was measured 48h later by qRT-PCR. CXCR4 mRNA levels were normalized for endogenous β-actin levels. The mean ± SE of three independent experiments, each in duplicate, is reported. ***, P = 0.001. PATZ1 and CXCR4 protein expression is shown on the top by western blot analysis, using vinculin detection as a loading control. Densitometric analysis by Image J software was applied: relative expression levels of CXCR4, compared to empty vector-transfected control and normalized with respect to vinculin, are indicated on the top of the blot.

Remarkably, low PATZ1 samples shown in Figure 6B and 6C were mostly characterized (11 out of 14) by increased expression of the G-protein coupled receptor CXCR4 (Supplementary Table 5), which has been shown to induce and assist in the maintenance of a mesenchymal expression profile in GBM cells [31] and is overexpressed in CD133+ cells from human GBMs resistant to chemotherapy [32]. Indeed, we found that CXCR4 was a high significant prognostic and predictive factor in proneural GBM, were high CXCR4 patients show worse outcome considering both OS and PFS survival curves (Figure 7A–7B), and negatively correlated with PATZ1 in these patients (r = -0.449; p = 0.03) (Figure 7C). A similar negative correlation between PATZ1 and CXCR4 was also observed in GSCs (Supplementary Figure 3).

In order to investigate a possible role of PATZ1 in downregulating *CXCR4* gene expression in GBM cells, we transiently transfected the VS-GB cells, a GBM-derived primary cell line that does not express PATZ1, with a plasmid expressing PATZ1, and measured *CXCR4* mRNA and protein levels by qRT-PCR and western blot, respectively (Figure 7D). A significant decrease of both mRNA and protein levels was observed in cells expressing PATZ1 compared to empty vector-transfected control, supporting an inhibitory role of PATZ1 on CXCR4 expression. This is consistent with data extracted from the Trafac homology server, showing that the CXCR4 promoter harbor consensus sequence for PATZ1 [33], and with data extracted from our previous analysis of Patz1-knockout mouse embryonic fibroblasts [34], showing that Patz1-null mouse embryonic fibroblasts express a 4-fold change increase of Cxcr4 compared to wild-type littermate controls.

## DISCUSSION

One of the most important hallmarks of GBM is the marked heterogeneity within and across these tumors, which has been attributed to cancer cell plasticity [35]. According to the cancer stem-cell model some cancers are organized into a hierarchy of subpopulations of tumorigenic cancer stem cells, which drive tumor growth, therapy resistance and disease progression. It is therefore imperative to clarify the markers that can be used to identify these cells and the context in which they work [36]. It has been recently proposed that patient-derived glioblastoma stem-like cells (GSCs) can be sub-divided in two main groups, one characterized by a proneural-like phenotype and the other showing a mesenchymal-like phenotype according to the molecular subtype classification of the TCGA [7–8]. Phenotypically, proneural GSCs exhibit non-adherent sphere forming growth *in vitro* and circumscribed, non-invasive growth *in vivo*. In contrast, mesenchymal cells grow semi-adherently *in vitro* and show invasive growth *in vivo* [8, 37]. Accordingly, patients with proneural GBM have a trend toward longer survival [7], whereas patients with mesenchymal GBM show the worst outcome [38].

In this study we analyzed expression of the Kruppel-type Zinc Finger transcription factor PATZ1 in GBM and oligodedrogliomas, finding it was highly expressed in a significant number of cases, whereas it was undetectable in normal glia. Moreover, we showed that PATZ1 is expressed in GSCs both *in vitro* (in GBM-derived stem cells) and *in vivo* (human tissue specimens), is downregulated in differentiated cells from the same tumor as compared to GSCs, and its expression is higher in GSCs growing as spheres (likely proneural) than in GSCs growing as adherent cells (likely mesenchymal). These observations were validated and completed in independent glioma datasets, finding a significant overlap of high PATZ1 expression with the proneural subtype and conversely of low PATZ1 expression with the mesenchymal subtype in both GBMs and GSCs. Since PATZ1 has been described as an inhibitor of cell proliferation in some cancer cell contexts [13,20,39], we would expect that GSCs growing as spheres that express higher levels of PATZ1 compared to GSCs growing in adhesion may proliferate less. However, since there are no differences in the proliferation rate between GSCs growing as spheres and those growing as adherent cells [26], we concluded that PATZ1 is not associated with proliferation in these cells. Interestingly, confocal microscopy analysis of human GBM specimens showed that PATZ1 was expressed in the NESTIN+ subpopulation, which has been recently shown to be crucial for tumor recurrence after treatment with the chemotherapeutic agent temozolomide [10].

Finally, we characterized the prognostic impact of PATZ1 expression first in our local cases of GBM, finding a significant worse outcome in patients showing absent or low levels of PATZ1 (< 10% positive cells), suggesting it may be used as a prognostic marker. Then, extending the analysis to the TCGA dataset, including samples subclassified in the four subtypes described by Verhaak et al. [7], the capacity of PATZ1 to discriminate GBM patients with different prognosis was significantly shown also in the proneural subtype, where low levels of PATZ1 correlate with worse outcome in both overall and progression-free survival curves, independently from other known prognostic factors, such as MGMT promoter methylation and age at diagnosis. Therefore, despite PATZ1 is enriched in the proneural subtype, there are proneural GBMs that express low levels of PATZ1 that are associated with a shorter patients survival. This is consistent with the already described intra-subtype heterogeneity of GBMs, which highlights the importance of finding new markers able to further sub-classify each subtype [35].

Since PATZ1 loss/downregulation has been associated with the acquisition of a mesenchymal phenotype in cancer cells [20], and the mesenchymal phenotype in GBMs is associated with the poorest prognosis and most aggressive phenotype, with advantage in proliferative capacity and neovascularization [5, 38], we speculate that a subset of proneural GBMs may be more aggressive due to the downregulation of PATZ1 and consequent activation of a mesenchymal program. Consistent with this hypothesis, we found that in the proneural subset there was a 75% of negative concordance (r = -0.44; p = 0.03) between PATZ1 and CXCR4, whose overexpression is an outcome common to multiple mechanisms of Epithelial-Mesenchymal Transition (EMT) induction in GBM and directly involved in determining the EMT phenotype [40]. Interestingly, according to the Trafac homology server, the CXCR4 promoter harbors consensus sequence for PATZ1 [33], and Patz1-null mouse embryonic fibroblasts express a 4-fold change increase of Cxcr4 compared to wild-type littermate controls [34]. Therefore, it is possible that PATZ1 could be directly involved in CXCR4 silencing. Indeed, it has been hypothesized that factors associated with the more stem-like phenotype may account for CXCR4 expression in GBM [27], and we provided evidence that PATZ1 could be one of these factors: in VS-GB cells, a primary GBM cell line that does not express endogenous PATZ1, overexpression of PATZ1 leads to downregulation of CXCR4. Further experiments are ongoing in our laboratory to explore the possibility of a direct role of PATZ1 on the CXCR4 promoter in GBM as well as in other cancer-derived cell lines, where PATZ1 appears involved in counteracting EMT. According to current opinions envisaging a parallel between dedifferentiation of cancer cells and reprogramming of somatic cells [41], the transdifferentiation of tumor cells from proneural to mesenchymal may imply a reprogramming process that leads differentiated cells to reacquire the capacity to differentiate into different cells. This is consistent with our previous results showing that PATZ1 plays an inhibitory role in the reprogramming process [34], and suggests a potential PATZ1 inhibitory role on the transition from the proneural to the mesenchymal phenotype. However, further functional experiments are needed to demonstrate the involvement of the PATZ1-CXCR4 axis in the proneural-to-mesenchymal phenotipic switch.

In conclusion, we describe PATZ1 as a novel biomarker of GBM and GSCs, differentially expressed among GBM and GSC subtypes and able to further subdivide the proneural GBM subtype in two populations with different overall and progression free survival. As PATZ1 has been proven an important transcription factor involved in stem cell pluripotency [12], it is encouraging to see that the proneural type of GBM, characterized by a stem-like signature, has higher expression of PATZ1 compared to other types. Finally, this finding may explain the paradox that the proneural type of GBM resists standard therapeutic caused by the cancer stem cells maintained by PATZ1 involved transcription factor networks, while at the same time PATZ1 restricts the proneural type of GBM to differentiation into mesenchymal type.

## MATERIALS AND METHODS

### Tissue specimens

45 patients diagnosed with glioblastoma and 22 with oligodendroglioma were included in the study. They had all undergone a complete excision of the tumor at first surgery. All the cases were identified between 2004 and 2013 and the diagnoses were made in accordance with the 2007 WHO classification [42]. This time interval was chosen in order to ensure a clinical follow-up period of at least 2 years for each identified case. All the cases were reviewed to evaluate adequate representative formalin-fixed paraffin-embedded tissue in order to perform immunohistochemical assay. We excluded those cases for which data of outcome were unavailable and for which diagnostic tissue was insufficient. Informed consent for the scientific use of biological material was obtained from all patients and the work has been approved by the local Ethical Committee (CEI 203/15 of 12/11/2015).

### Immunohistochemical analysis

4 μm sections were used for immunohistochemistry. Sections were dewaxed in xylene, hydrated in graded series of alcohol and subjected to heat-induced antigen retrieval. After blocking endogenous peroxidase activity, the tissue was incubated with antibody anti-PATZ1 (polyclonal rabbit, R1P1 [22]; 1: 500 dilution, overnight incubation). Subsequently, the slices were rinsed and incubated with the biotinylated secondary antibody, at room temperature, for 30 minutes. The bound antibody complexes were stained for 3-5 minutes or until appropriate for microscopic examination with diaminobenzidine, and slides were then counterstained with hematoxylin (30s) and mounted. All slices were reviewed by two experienced pathologists (M.L.D., E.G.).

The assessment of PATZ1 (nuclear) staining was based on the percentage of positive cells: no signal was set as “0”, ≤10% as “+”, >10% and <50% as “++” and ≥50% as “+++”. We defined high expression of PATZ1 as >10% of cells staining positive, and low expression as ≤10% of cells staining positive.

### Immunofluorescence and confocal microscopy

For multiple-labeling immunofluorescence, tissues slides were dewaxed, hydrated and antigen retrieval was performed using Dewax and HIER Buffer L (Thermo Fisher Scientific, Cheshire, UK) as previously described [43]. They were incubated with the mixture of two primary antibodies anti-NESTIN (mouse monoclonal, sc23927, Santa Cruz Biotechnology, Santa Cruz, CA; 1:200 dilution, 90 minutes incubation) and anti-PATZ1 (rabbit polyclonal, R1P1; 1: 500 dilution, overnight incubation) or anti PATZ1 and anti-OCT-3/4 (mouse monoclonal, sc-5279 Santa Cruz Biotechnology; 1:100 dilution, 90 minutes incubation) in 1% BSA in PBS-T in a humified chamber overnight at 4°C. Fluorescent secondary antibodies, raised against different species (Alexa Fluor 594, with red signal, against rabbit and Alexa Fluor 488, with green signal, against mouse), were used to locate the antigen/primary antibody complexes. Counter staining was made with Hoechst 33258 (nuclear blue staining) present in mounting medium (PBS:Glycerol = 1:1). Experiments were carried out on an inverted and motorized microscope (Axio Observer Z.1) equipped with a 63x/1.4 Plan-Apochromat objective. The attached laser scanning unit (LSM 700 4x pigtailed laser 405-639, Zeiss, Jena, Germany) enabled confocal imaging. For excitation, 405 nm, 488 nm and 555 nm lasers were used. Fluorescence emission was revealed by MBS 405/488/555/639 for Hoechst 33258, Alexa Fluor 488 and Alexa Fluor 594. Triple staining immunoflourescence images were acquired separately in the blue, green and red channels at a resolution of 1024×1024 pixels, with the confocal pinhole set to one Airy unit and then saved in TIFF format.

To assess the stemness of GSCs, NANOG (rabbit polyclonal, ab80892 Abcam; 1:200 dilution), SOX-2 (mouse polyclonal, AB5603 Millipore; 1:500 dilution), OCT-4 (rabbit polyclonal, sc-5279 Santa Cruz; 1:250 dilution) and NESTIN (mouse monoclonal, 92590 Chemicon; 1:100 dilution) expression was evaluated. PATZ1 (rabbit polyclonal, R1P1, 1:500 dilution) expression was analyzed as well. Briefly, cells were fixed in 4% paraformaldehyde for 20 minutes at room temperature (RT) and permeabilized for 10 minutes at RT with 0.1% Triton X-100 (Sigma-Aldrich) before exposing them to primary antibodies, that were incubated over-night at 4°C. Subsequently, Alexa Fluor 488 and Alexa Fluor 555 dyes labeled secondary antibodies against mouse and rabbit, respectively, diluted 1:800 in PBS, were employed for 1 hour in a humidified chamber at 37°C (Molecular Probe, Invitrogen). Lastly, slides were mounted using Vectashield (Vector), as mounting medium, added with 0.1 μg/ml DAPI (Sigma) for nuclear staining. Images were acquired utilizing a live cell imaging dedicated system consisting of a Leica DMI 6000B microscope connected to a Leica DFC350FX camera (Leica Microsystems, Wetzlar, Germany) and equipped with a 63X/1.4 oil immersion objective or a 40X/1.25 oil immersion objective. Adobe Photoshop software was utilized to compose and overlay the images and to adjust contrast.

### Cell cultures and transfection

The independent ethic committee of the Azienda Ospedaliero-Universitaria of Udine has approved the research (Consents 102/2011/Sper and 196/2014/Em). Informed written consents were obtained from patients and all clinical investigations have been conducted according to the principles expressed in the Declaration of Helsinki.

GSCs (n=11) were isolated as previously described [44,45]. Briefly, human GBM samples were mechanically-enzymatically dissociated and cells less than 40μm in diameter cultured on laminin-coated dishes in a growing medium composed as follows: Neurobasal-A medium (Gibco by Invitrogen, Carlsbad, CA), 2mM L-glutamine (Sigma-Aldrich, St. Louis, MO,), 1X N2 supplement (Gibco by Invitrogen), 25mg/ml Insulin, Penicillin-streptomycin, 100mg/ml human apo-trasferrin (Sigma-Aldrich), 1X B-27 supplement (Gibco by Invitrogen), 20 ng/ml recombinant human basic fibroblast growth factor (h-FGF-basic) (Peprotech, Rocky Hill, NJ), 20 ng/ml human epidermal growth factor (h-EGF) (Peprotech). Then cells were cultured in an incubator at 37°C, 5%O2/5%CO2 and analyzed at the third passage in culture. From six GBM samples we isolated, in addition to GSCs, the matched non-stem tumor cells by growing dissociated cells in DMEM (Gibco by Invitrogen) supplemented with 10% fetal bovine serum (FBS) (EuroClone, Pero, Italy) and 100 U/ml Penicillin 100 μg/ml Streptomycin. Cells were grown in an incubator at 37°C, 5%O2/5% CO2 and analyzed at the first passage in culture.

Human GBM U87MG (American Type Culture Collection, ATCC no. HTB-14), were grown in Dulbecco's modified Eagle medium (DMEM) supplemented with 10% FBS. To obtain tumorspheres, U87MG cells were grown at low density (2000 cells/ml) in Stem cell medium (SM) [DMEM-F12 supplemented with 1% B27 (Sigma-Aldrich), 10 ng/ml h-FGF (Sigma-Aldrich) and 20 ng/ml h-EGF (Sigma-Aldrich)] for 12 days. U87MG tumorspheres were induced to differentiate through the addiction of 10% FBS in SM medium. The cancer stem cell phenotype of these cells was confirmed by assessing the expression of the stemness markers PDGFRß, NANOG and SOX-2.

VS-GB primary cell culture was derived from GBM specimens and grown as described previously [46]. They were transiently transfected by JetPRIME (Polyplus transfections, Illkirch, France) with 10 μg of HA-PATZ1 plasmid encoding for PATZ1 variant 4 or 10 μg of the empty vector pCEFL-HA as negative control, according to manufacturer's instruction.

### RNA extraction and quantitative real time (qRT)-PCR

Total RNA from cells was purified by TRIzol (Invitrogen). After digestion of the residual genomic DNA by DNase I (Qiagen, Milano, Italy), RNA was reverse transcribed by SuperScript III cDNA synthesis kit (Invitrogen). cDNA was used to perform qPCR reactions with SYBR Green Master Mix gene expression kit (Biorad, Segrate, Italy) in 96-well plates with a LightCycler 480 system (Roche, Monza, Italy). The PCR conditions were as follow: 95°C for 3 minutes; 45 cycles at 95°C for 10 seconds, 60°C for 20 seconds and 72°C for 60 seconds. Primer pairs used (300 nm each) were as follows: *PATZ1* (5’-TACATCTGCCAGAGCTGTGG-3’/5’-TGCACCTGCTTGATATGTCC-3’); *GAPDH* (5’- GTCTCCTCTGACTTCAACAGCG-3’/5’-ACCACCCTG TTGCTGTAGCCAA-3’). *CXCR4* (5’-TGAGAAG CATGACGGACAAG-3′/5’-AGGGAAGCGTGATGAC AAAG-3′); *β-ACTIN* (5’-CAAGAGATGGCCAC GGCTGCT-3’/5’-TCCTTCTGCATCCTGTCGGCA-3’). Gene expression changes were calculated using the 2(-Delta Delta C(T)) method [47].

### Protein extraction and western blot analysis

Cells were lysed in buffer containing 1% Nonidet P-40, 1 mmol/liter EDTA, 50 mmol/liter Tris-HCl (pH 7.5), and 150 mmol/liter NaCl supplemented with Complete protease inhibitors (Roche). Total proteins were resolved in a 10-12% polyacrylamide gel under denaturing conditions and transferred to nitrocellulose filters for Western blot analyses. Membranes were blocked with 5% BSA in TBS and incubated with the primary antibodies. Membranes were then incubated with the horseradish peroxidase–conjugated secondary antibody (1:3.000) and the reaction was detected with a Western blotting detection system (enhanced chemiluminescence; GE Healthcare). The primary antibodies used were: anti-PATZ1 [22]; anti-NESTIN (sc-23927, Santa Cruz Biotechnology, Santa Cruz, CA); anti-PDGFRβ (3169, Cell Signaling Technology, Boston, MA); anti-NANOG (sc-134218, Santa Cruz Biotechnology); anti-SOX2 (sc-20088, Santa Cruz Biotechnology); anti-CXCR4 (C8727, Sigma-Aldrich). To ascertain that equal amounts of protein were loaded, the membranes were incubated with anti-ACTIN (sc-1616-R, Santa Cruz Biotechnology) or anti-VINCULIN (sc-7649, Santa Cruz Biotechnology) antibodies.

### Data mining

For PATZ1 mRNA expression analysis and correlation with clinical, molecular and cell phenotype, the Genomics Analysis and Visualization platform (http://r2.amc.nl) was used with the following datasets: 1) GSE9385 [25], including 6 adult non diseased brain, 26 glioblastomas and 21 oligodendrogliomas,; 2) GSE8049 [26], including 9 different glioblastoma-derived neurosphere cultures in two biological replicates; 3) GSE15209 [28], including stem cells derived from 7 GBMs, 2 oligoastrocytomas, 5 normal fetuses and primary biopsies from 6 normal brains; 4) GSE18015 [29], including 8 gliomas sorted for CD133 expression; 5) the TCGA dataset Tumor Glioblastoma - 540 - MAS5.0 - u133a (n = 540) (http://cancergenome.nih.gov), including 85 samples sub-classified in classical (n = 17), mesenchymal (n = 27), neural (n = 17) and proneural (n = 24) GBM [7], to correlate *PATZ1* expression to GBM subtype and specific gene signatures. The last dataset was also used for Overall and Progression-free survival analysis using the scan cut-off mode based on median PATZ1 expression and specifying the proneural track subset.

### Statistical analysis and Kaplan-Meier survival curves

The Fisher's exact test was used to evaluate the correlations between PATZ1 score and the clinical variables (age, gender, site, oligodendroglioma grading).

All correlations within the TCGA and other public datasets were assessed by Pearson's χ^2^ test or one-way analysis of variance (ANOVA), through the R2 platform (http://r2.amc.nl).

Kaplan-Meier survival curves were used to analyze OS and PFS, and statistical significance was assessed by the log-rank test. In Figure 6A, the stratification of High/Low PATZ1 was based on the percentage of cells expressing nuclear PATZ1 protein by immunohistochemistry (High: > 10%; Low: < 10%). The stratification of High/Low *PATZ1* and High/Low *CXCR4* in the TCGA dataset was based on RNA expression using a cutoff of 100.6 and 197.7, according to the scan function of the R2 platform used, where an optimum survival cutoff is established based on statistical testing (log-rank test).

PATZ1 expression on human derived stem cells was expressed as mean ± standard deviation. Comparisons between two groups were performed by paired or unpaired t test, as appropriate.

All statistical analyses were performed using GraphPad Prism 5 software (GraphPad Software, La Jolla, CA). A probability (p) value less than 0.05 was considered statistically significant.

## SUPPLEMENTARY MATERIALS FIGURES AND TABLES



## Abbreviations

GBM: glioblastoma
GSCs: glioma-initiating stem cells
TCGA: The Cancer Genome Atlas
PATZ1: POZ, AT-hook and zinc-finger 1
OS: overall survival
PFS: progression-free survival
EMT: epithelial-mesenchymal transition
RT: room temperature
h-FGF-basic: human basic fibroblast growth factor
h-EGF: human epidermal growth factor
FBS: fetal bovine serum
DMEM: Dulbecco's modified eagle medium
SM: stem cell medium
IHC: immunohistochemistry.
