# Supplementary material for: PATZ1 is a new prognostic marker of glioblastoma associated with the stem-like phenotype and enriched in the proneural subtype
Source: Oncotarget. 2017 Jul 25;8(35):59282–300. doi: 10.18632/oncotarget.19546 (PMC5601732; doi:10.18632/oncotarget.19546)
Supplement: Supplementary file 1 [file oncotarget-08-59282-s001.pdf]

PATZ1 is a new prognostic marker of glioblastoma associated with the stem-like phenotype and enriched in the proneural subtype

SUPPLEMENTARY MATERIALS

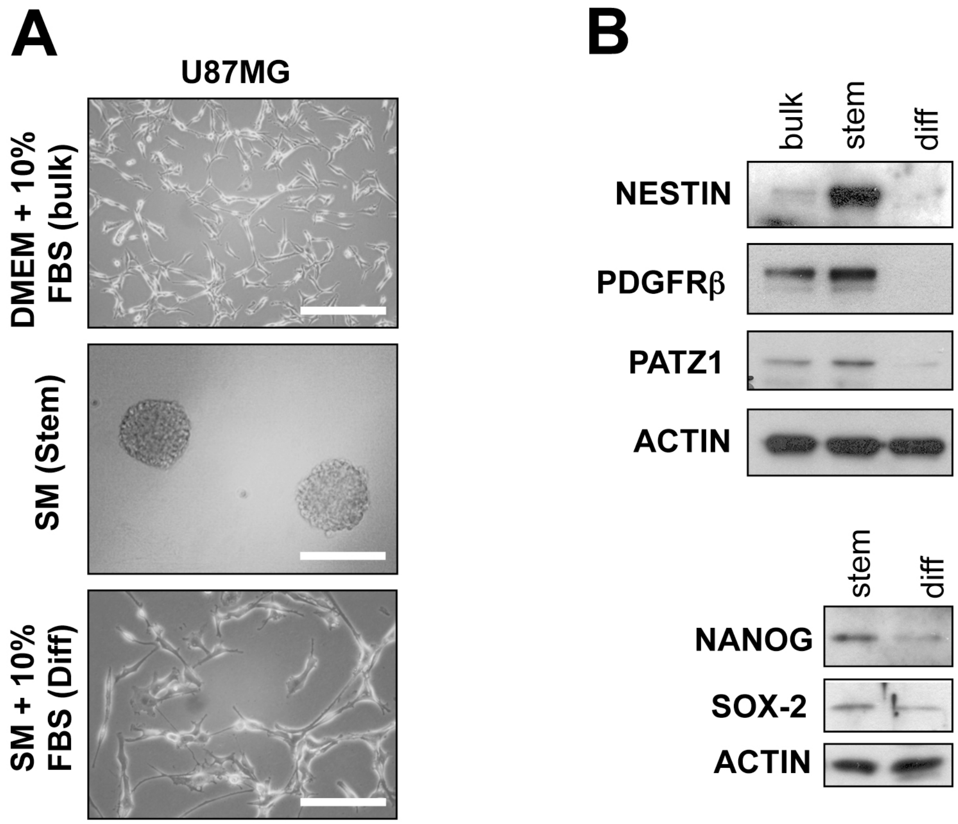

Supplementary Figure 1: (A) Representative microscope images of U87MG cells (bulk), isolated U87MG spheres grown in stem cell medium (SM) without (Stem) or with (Diff.) 10% FBS. Scale bar: 200 μm (upper panel), 100 μm (middle and lower panels). **(B)** Western bolt analysis of cells shown in A. Proteins detected are indicated on the side. Actin was evaluated as a loading control.

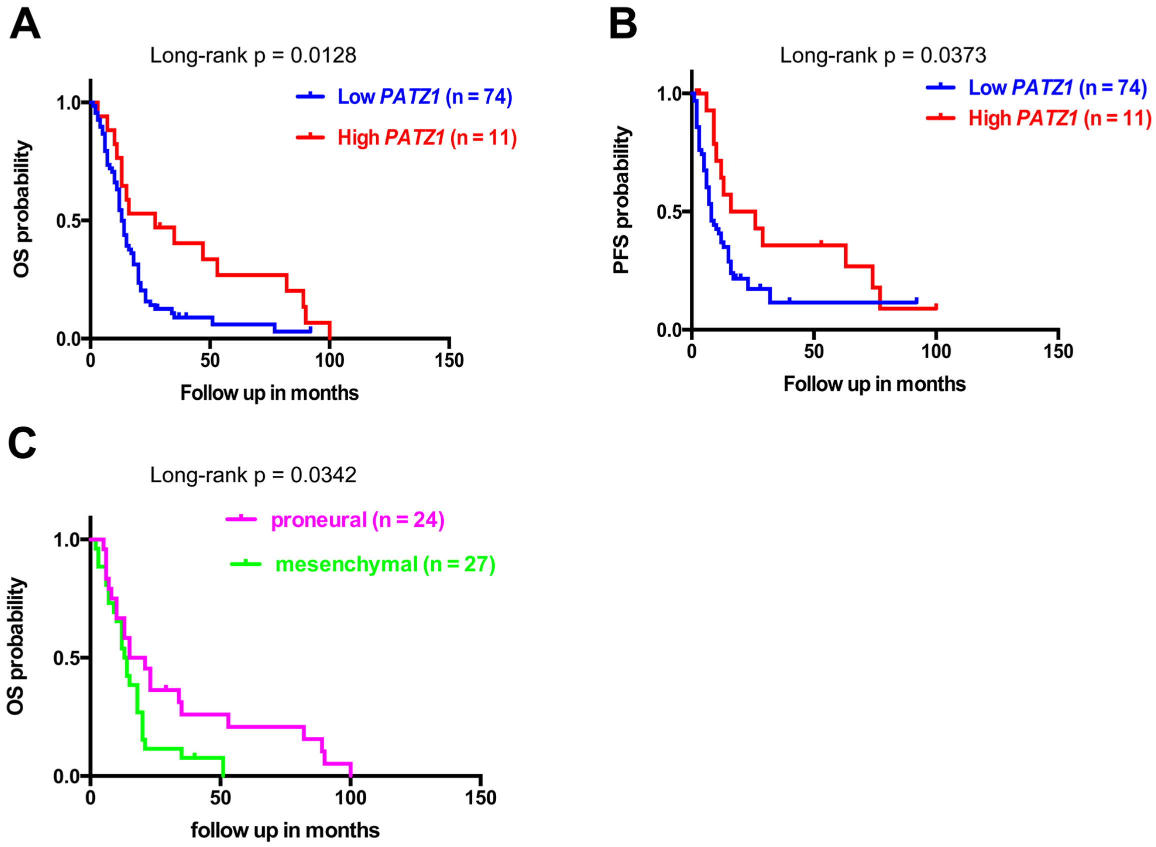

Supplementary Figure 2: (A) Overall (OS) and **(B)** progression-free (PFS) Kaplan-Meier curves of GBM patients from the TCGA data set already shown in Figure 3B, including 85 samples representative of the four GBM subtypes. Note that patients with lower PATZ1 expression have a worse survival rate than patients with higher PATZ1 as assessed by log-rank test. **(C)** Overall survival in proneural and mesenchymal GBM subtypes of the same data set.

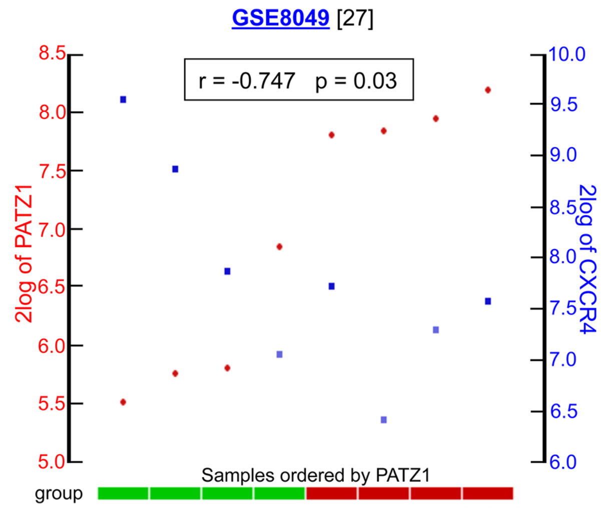

Supplementary Figure 3: YY-plot correlating PATZ1 and CXCR4 expression in GSCs, including 4 of the restricted Gsr phenotype (green) and 4 of the full Gsf phenotype (red). Significance of correlation (inbox) was analyzed by Pearson's χ^2 test through the R2 web platform (http://r2.amc.nl).

Supplementary Table 1: Clinicopathological features and PATZ1 expression of 45 glioblastomas (local cohort analyzed by IHC)

Case	Gender	Age	Site	PATZ1
1	F	71	f	-
2	F	62	t	-
3	M	70	t	+
4	F	66	t	++
5	M	35	p	++
6	M	65	t	-
7	F	69	t	-
8	F	41	f	+
9	F	58	f	+
10	M	64	t	+++
11	F	64	t	++
12	M	72	t	+
13	M	58	f	+
14	M	74	t	+
15	M	62	t	-
16	F	44	c	-
17	F	64	p	-
18	M	62	t	++
19	M	60	t	+
20	M	65	f	-
21	M	57	f	+
22	M	66	p	++
23	F	36	t	++
24	M	62	o	+
25	M	62	t	+
26	M	63	o	+
27	M	69	t	+++
28	F	26	p	++
29	M	73	t	-
30	F	72	t	++
31	M	73	p	+++
32	M	70	p	-
33	M	52	t	+++
34	M	41	t	++
35	M	63	p	++
36	M	60	p	++
37	M	61	t	-
38	M	64	p	+
39	M	69	p	+++
40	F	81	p	+
41	F	75	f	-
42	M	54	f	+
43	M	68	t	+
44	M	74	f	+
45	F	72	t	-

f, frontal; t, temporal; o, occipital; p, parietal; c, corpus callosum

Supplementary Table 2: Clinicopathological features and PATZ1 expression of 22 oligodendrogliomas (local cohort analyzed by immunohistochemistry)

Case	Gender	Age	Site	Grade	PATZ1
1	M	66	t	III	+
2	F	67	f	II	-
3	M	44	f	II	-
4	F	50	t	II	-
5	F	54	f	III	-
6	M	39	f	II	-
7	M	57	o	III	+
8	M	33	f	II	+++
9	M	26	f	II	-
10	M	58	o	III	+
11	M	31	f	II	-
12	M	35	f	II	++
13	F	54	t	III	++
14	M	69	f	III	++
15	M	69	o	III	++
16	F	38	t	III	-
17	M	41	i	III	+++
18	M	41	f	III	+++
19	F	43	f	II	++
20	F	40	f	II	+
21	M	77	f	II	-
22	M	22	f	II	-

f, frontal; t, temporal; o, occipital; p, parietal; i, intramedullary metastasis

Supplementary Table 3: Other correlations between *PATZ1* and the mesenchymal signature in GBM[a]

Gene	r[b]	p	gene	R	P
THBS1	-0.317	3.2e-13	*DAB2*	-0.270	8.1e-10
COL8A2	-0.317	3.4e-13	*TGOLN2*	-0.265	1.9e-09
LTBP1	-0.314	5.8e-13	*NRP1*	-0.262	2.9e-09
HFE	-0.305	2.8e-12	*LAPTM5*	-0.262	2.8e-09
LCP1	-0.305	3.1e-12	*NCF4*	-0.253	9.8e-09
TNFRSF1A	-0.302	5.1e-12	*COL1A2*	-0.251	1.5e-08
PDPN	-0.298	8.8e-12	*DSC2*	-0.250	1.6e-08
ADAM12	-0.297	1.2e-11	***CD44***	-0.242	4.6e-08
TNFRSF1B	-0.297	1.1e-11	*EFEMP2*	-0.241	5.6e-08
IL15RA	-0.294	1.9e-11	*COL5A1*	-0.241	5.2e-08
PLK3	-0.290	3.9e-11	*WWTR1*	-0.237	8.8e-08
ITGA5	-0.289	4.5e-11	*PTRF*	-0.236	1.0e-07
SLC11A1	-0.285	8.0e-11	*MVP*	-0.234	1.4e-07
TRADD	-0.280	1.8e-10	*PTPN6*	-0.231	2.0e-07
MAPK13	-0.278	2.4e-10	*MRC2*	-0.231	2.0e-07
CDCP1	-0.277	3.0e-10	*IL4R*	-0.228	3.0e-07
RAB11FIP1	-0.277	2.8e-10	*CDAP*	-0.225	4.6e-07
CLCF1	-0.276	3.6e-10	*HK3*	-0.223	5.6e-07
GCNT1	-0.276	3.2e-10	*FNDC3B*	-0.212	2.1e-06
LCP2	-0.275	4.2e-10	*DCBLD2*	-0.210	2.6e-06
FOLR2	-0.274	4.4e-10	*CNN2*	-0.207	3.9e-06
TNFRSF11A	-0.271	7.7e-10			

[a] All genes belong to the mesenchymal signature described by Verhaak et al. [7]

[b] Correlations were analyzed by Pearson's χ^2 test through the R2 platform (http://r2.amc.nl)

Supplementary Table 4: Oligodendrocyte precursor and proneural gene expression correlations with PATZ1 in GBM-derived stem cells

	GSE8049 (Gunter et al. 2008)	GSE15209 (Pollard et al. 2009)
DLL3	r = 0.844; p = 2.1e-05	r = 0.56; p = 1e-02
SOX2	r = 0.617; p = 8.4e-03	r = 0.837; p = 4.2e-06
SOX8	r = 0.681; p = 2.6e-03	r = 0.704; p = 5.3e-04
NES	r = 0.655; p = 4.3e-03	r = 0.611; p = 4.2e-03
OLIG2	r = 0.739; p = 7.1e-04	r = 0.556; p = 1e-02
OLIG1	r = 0.687; p = 2.3e-03	r = 0.556; p = 1e-02
NKX2.2	r = 0.566; p = 2e-02	r = 0.494; p = 3e-03
DCX	r = 0.488; p = 5e-02	r = 0.705; p = 5.2e-04
ASCL1	r = 0.761; p = 3.9e-04	r = 0.778; p = 5.3e-05
TCF4	r = 0.577; p = 2e-02	r = 0.582; p = 7.1e-03

Supplementary Table 5: Molecular and clinical features in proneural GBM (TCGA dataset)

ID patient	OS f/u (months)	PFS f/u (months)	Age	MGMT methylation	*PATZ1* (Cutoff: 100.6)	*CXCR4* (Cutoff: 197.7)
02-0003[a]	5	5	50	No	Low	High
06-0648	10	7	77	Yes	Low	High
12-0618	13	2	49	No	High	High
06-0238	13	10	46	No	High	High
12-0616	15	13	36	No	High	High
02-0069	29	29	31	Yes	High	Low
02-0010	35	12	20	No	High	Low
02-0024	53	53	35	No	High	Low
02-0014	82	74	25	No	High	Low
02-0080	89	77	28	No	High	Low
02-0028	90	63	39	Yes	High	Low
02-0114	100	100	37	Yes	High	Low
06-0646	6	3	60	No	Low	High
06-0166	6	2	51	No	Low	High
02-0060	6	6	66	No	Low	High
02-0046	7	6	61	No	Low	High
02-0058	8	6	28	Yes	Low	High
02-0074	10	5	68	No	Low	High
06-0241	15	15	66	Yes	Low	Low
02-0047	15	2	78	No	Low	High
02-0011	21	5	19	No	Low	Low
06-0128	23	6	66	No	Low	High
02-0007	23	17	40	No	Low	High
06-0129	34	5	30	No	Low	Low

[a] Samples highlighted in gray show opposite expression levels of *PATZ1* and *CXCR4* (75% negative concordance)
